# PI3 Kinase Pathway and MET Inhibition is Efficacious in Malignant Pleural Mesothelioma

**DOI:** 10.1038/srep32992

**Published:** 2016-09-13

**Authors:** Rajani Kanteti, Jacob J. Riehm, Immanuel Dhanasingh, Frances E. Lennon, Tamara Mirzapoiazova, Bolot Mambetsariev, Hedy L. Kindler, Ravi Salgia

**Affiliations:** 1 Department of Hematology/Oncology, University of Chicago Medical Center, Chicago, IL, USA

## Abstract

Malignant pleural mesothelioma (MPM) is an aggressive cancer that is commonly associated with prior asbestos exposure. Receptor tyrosine kinases (RTKs) such as MET and its downstream target PI3K are overexpressed and activated in a majority of MPMs. Here, we studied the combinatorial therapeutic efficacy of the MET/ALK inhibitor crizotinib, with either a pan-class I PI3K inhibitor, BKM120, or with a PI3K/mTOR dual inhibitor, GDC-0980, in mesothelioma. Cell viability results showed that MPM cells were highly sensitive to crizotinib, BKM120 and GDC-0980 when used individually and their combination was more effective in suppressing growth. Treatment of MPM cells with these inhibitors also significantly decreased cell migration, and the combination of them was synergistic. Treatment with BKM120 alone or in combination with crizotinib induced G2-M arrest and apoptosis. Both crizotinib and BKM120 strongly inhibited the activity of MET and PI3K as evidenced by the decreased phosphorylation of MET, AKT and ribosomal S6 kinase. Using a PDX mouse model, we showed that a combination of crizotinib with BKM120 was highly synergetic in inhibiting MPM tumor growth. In conclusion our findings suggest that dual inhibition of PI3K and MET pathway is an effective strategy in treating MPM as compared to a single agent.

Malignant mesothelioma (MM) is a slow growing, solid tumor that primarily originates in pleural (80%), peritoneal (20%) and pericardial cavities (1%)^1^,^2^. Various etiological factors contribute to the onset of MM such as exposure to asbestos or erionite, Simian virus 40 (SV40), genetic predisposition and radiation therapy^3^,^4^,^5^. The current standard therapy for MM consists of surgical resection, combination chemotherapy with cisplatin and pemetrexed, and potentially radiation^6^,^7^. Despite advances in chemotherapy, MM has very poor prognosis and median survival of less than one year which is unacceptably low^8^. Therefore, there is a pressing need for more efficacious therapies for MM.

RTKs are known to play a crucial role in tumor growth and metastasis. Some RTKs were originally discovered as oncogenes and are known to provide key signals that lead to transformation, tumor growth and metastasis^9^. Several studies have demonstrated that RTKs including epidermal growth factor receptor (EGFR), MET, insulin growth factor receptor (IGFR) and vascular endothelial growth factor receptor (VEGFR) are overexpressed in MPM^10^,^11^,^12^,^13^.

Previously we demonstrated that the MET/HGF axis is activated in MPM through overexpression, amplification and mutations of MET. SU11274, a small molecule inhibitor of MET is known to decrease cell proliferation of mesothelioma cells^14^.

Crizotinib (PF02341066, Pfizer) is an orally available, potent, ATP competitive, small molecular inhibitor of MET, anaplastic lymphoma kinase (ALK) and c-Ros Oncogene 1 (ROS1). Its affinity for MET is greater than that for ALK or ROS1. FDA has approved its use for the treatment of NSCLC^15^.

Phosphatidylinositol 3-kinase (PI3K) is a key downstream signaling molecule of MET and other RTKs. It is a cellular proto-oncogene and an essential lipid kinase, that plays an important role in the regulation of cell proliferation, survival and motility^16^. Several preclinical studies have shown that this pathway is hyper activated in mesothelioma^17^,^18^.

BKM120 is a potent inhibitor of class I PI3Ks, currently in Phase I and II clinical trials for patients with a variety of solid tumors. Another PI3K inhibitor we investigated in this study is GDC-0980, a potent small molecule inhibitor of class I PI3K isoforms and mTOR.

In the present study, we have investigated the effects of crizotinib and BKM120, singly or in combination, on MPM tumor growth using both *in vitro* and *in vivo* models. Apart from BKM120 similar results were also observed with GDC-0980. While single use of BKM120 inhibited growth of MPM tumor in a PDX mouse model, the combined treatment with crizotinib and BKM120 was highly synergistic.

## Results

### Synergistic suppression of MPM cell proliferation using MET and PI3K inhibitors

**Figure 1:**
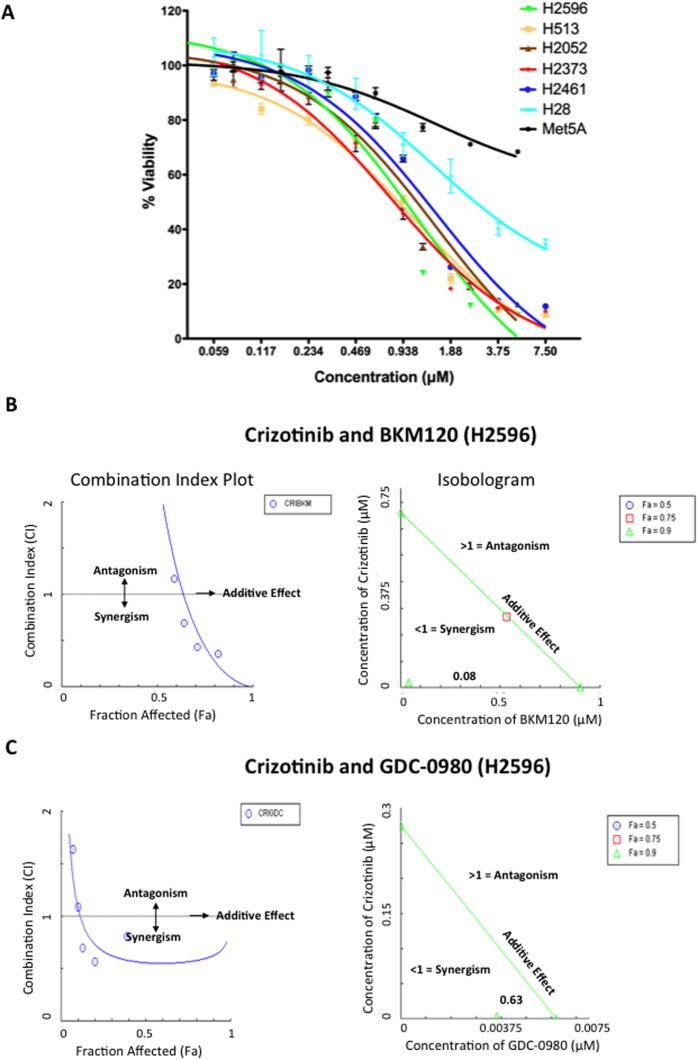
Effect of MET and PI3K inhibitors on proliferation of human mesothelioma cells and Synergistic anti-tumor activity of these inhibitors. (**A**) Mesothelioma cell lines H2373, H2596, H513, H2052, H2461, H28, and Met-5A were treated with BKM120 for 72 h. Viability was measured by Alamar Blue assay. The data shown represents the average ± SEM. (**B**) Combination Index plot and Isobologram for combination of Crizotinib and BKM120 in H2596 cells. The left side panel shows the CI plot for the combinations of drugs where synergy (identified by a Combination Index <1) over a range of drug concentrations. The green triangle in the isobologram represents concentrations of both drugs that inhibit cellular proliferation by 90% (Fraction affected = 0.9). A combination index (CI) value of 0.08 was calculated using CompuSyn software. The line represents an additive affect, where CI = 1. (**C**) Combination Index plot and Isobologram for combination of Crizotinib and GDC-0980 in H2596 cells. The left side panel shows the CI plot for the combinations of drugs where synergy (identified by a Combination Index <1) over a range of drug concentrations. The green triangle in the isobologram represents concentrations of both drugs that inhibit cellular proliferation by 90% (Fraction affected = 0.9). A combination index (CI) value of 0.63 was calculated using CompuSyn software. The line represents an additive affect, where CI = 1.

Cell viability was determined following treatment with increasing concentrations of BKM120 for 72 h and results are presented in Fig. 1. Most of the MPM cell lines used were sensitive to treatment with BKM120 with IC_50_ values ranging from 0.79–1.51 μM. However, Met-5A, a control mesothelial cell line and H28 were less sensitive to BKM120 (Fig. 1A).

The cells were treated with a combination of crizotinib and BKM120 for 72 h and the viability was determined. This combination had a significant synergistic effect on the suppression of growth of H2596 (Fig. 1B) and H513 cell lines (Supplemental Fig. 1A). Drug synergy here is measured by isobologram and fraction Combination index plot, which are the two robust methods for evaluating drug interactions in combination cancer chemotherapy. Isobolograms provide a visual approach to assessing the possibility of synergy. The points on the axes represent the IC_50_ values for either crizotinib alone or BKM120/GDC-0980 alone. Combination drug points falling on or around the line connecting the IC_50_ values alone represent additivity, while points falling below the line suggest synergy. Isobologram analysis provides a combination index (CI) value, which ultimately measures the synergy. The combination-index (CI) method is a mathematical and quantitative representation of a two-drug pharmacologic interaction. Since both the graphics are based on the same combination index equation, they give the same conclusion. Next, the effect of the combination of crizotinib with GDC-0980 (Fig. 1C) was determined in H2596 (Fig. 1C) and H2052 (Supplemental Fig. 1B) cells and found to be also synergistic in suppressing growth of MPM cells. The complete Compusyn Reports have been included in the Supplementary Datasets 1–4. The growth of MPM cell lines, except for Met-5A, were found to be sensitive to the MET inhibitor crizotinib^19^.

### Combined use of MET and PI3K inhibitors suppresses cell migration

**Figure 2:**
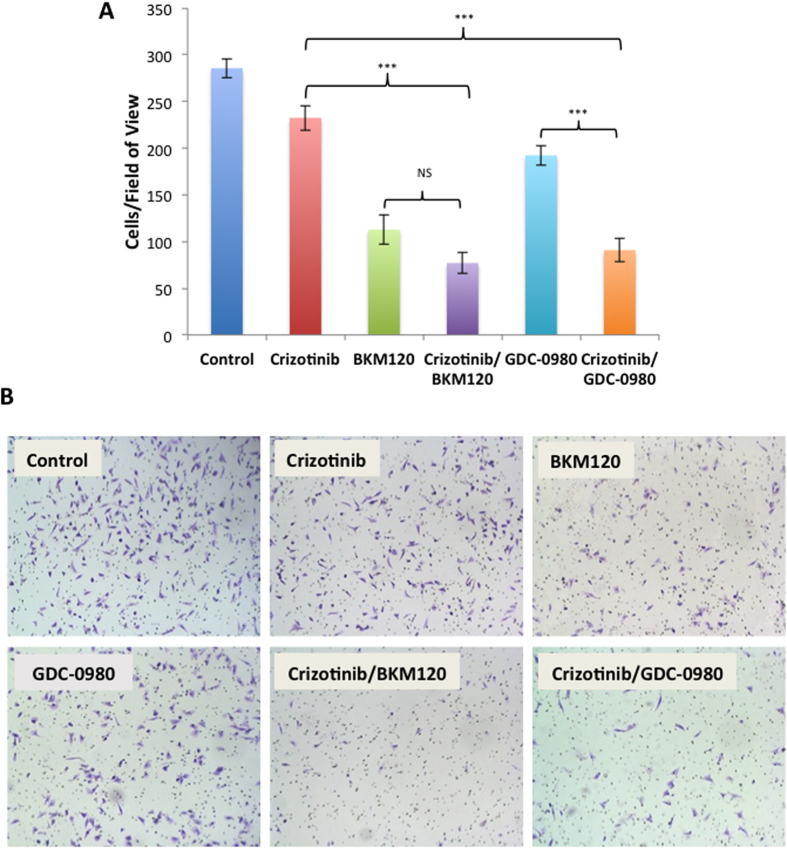
Effect of MET and PI3K inhibitors on MPM cell migration. The H2596 cells were treated with 1 μM crizotinib, 2 μM BKM120, 0.25 μM GDC-0980 alone and in the indicated combinations for 16 h. (**A**) The migrated cells were counted using ImageJ. The experiments were done in triplicate and data is shown as bar graphs. Results were analyzed by ANOVA with Tukey post-test. (NS p > 0.05, *p < 0.05, **p < 0.01, ***p < 0.001). (**B**) Representative images of the migrated cells stained using crystal violet are shown.

Next the effect of MET and PI3K inhibitors on migration of MPM cells was determined using Boyden chamber migration assay with fetal bovine serum as the chemo attractant. Treatment of MPM cells with either, crizotinib, BKM120 or GDC-0980 significantly decreased migration of cells compared to control cells (crizotinib p < 0.05, BKM120 and GDC-0980 p < 0.001). The combination of crizotinib with either BKM120 or GDC-0980 further decreased cell migration and was significantly synergistic compared to single drug treatment with crizotinib or GDC-0980 (crizotinib Vs BKM120/crizotinib p < 0.001 and GDC-0980 Vs crizotinib/GDC-0980 p < 0.001). Treatment with BKM120/crizotinib combination did further inhibit the migration compared to BKM120 alone, but the decrease was not statistically significant. (p > 0.05) (Fig. 2A,B).

### Combined use of MET and PI3K inhibitors induces cell cycle arrest and activate apoptotic pathways

**Figure 3:**
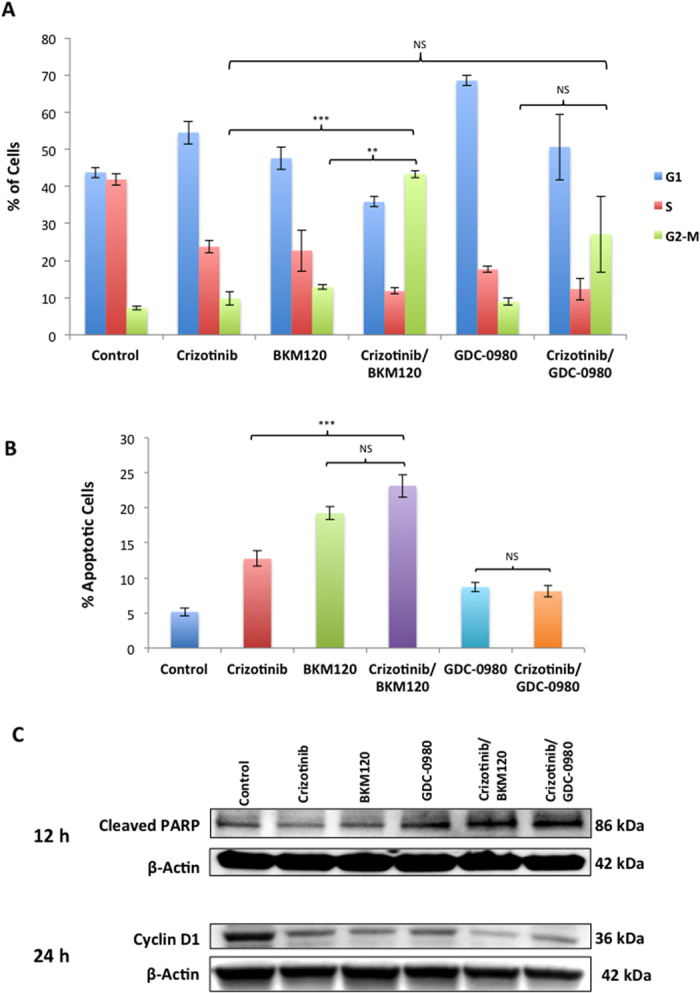
Crizotinib, BKM120, and GDC-0980 alone or in combination induce cell cycle arrest and apoptosis. (**A**) Summary of the percentage of cells in each cell cycle phase after the treatment of MPM cells with 1 μM crizotinib, 2 μM BKM120, 0.25 μM GDC-0980 alone and in the indicated combinations for 48 h. Data is shown as the % of cells in G1, S and G2-M phases ± SEM. Results were analyzed by ANOVA with Tukey post-test. (NS p > 0.05, *p < 0.05, **p < 0.01, ***p < 0.001). (**B**) H2596 cells were treated with 1 μM crizotinib, 2 μM BKM120, 0.25 μM GDC-0980 alone and in the indicated combinations for 48 hours then stained with Annexin V-FITC/PI and analyzed by flow cytometry. Results are expressed as the mean percentage of apoptotic cells ± SEM. (**C**) Cells, were treated with 1 μM crizotinib, 2 μM BKM120, 0.25 μM GDC-0980 alone and in the indicated combinations for 12 h and 24 h. Cell lysates were prepared and immunoblotted for cleaved PARP, cyclin D1 antibodies and actin was used as a loading control.

In order to determine the underlying mechanisms related to the loss of cell viability induced by the inhibitors in these cells, the effects on cell cycle progression were investigated. After 48 h treatment various cell cycle phases were analyzed by flow cytometry. Treatment of H2596 cells with crizotinib, BKM120 and GDC-0980 alone had no effect on G2-M arrest in cell cycle. However the combination of BKM120/crizotinib significantly increased the percentage of cells arrested in G2-M phase (crizotinib Vs BKM120/crizotinib p < 0.001) (BKM120 Vs BKM120/crizotinib p < 0.01) (Fig. 3A).

The effect of the above inhibitors on apoptosis was next determined as growth arrest of cancer cells, especially in G2-M phase is known to trigger apoptosis. H2596 cells were treated with inhibitors for 48 h and apoptosis was measured by Annexin V staining. While treatment of H2596 cells with crizotinib alone resulted in a 2.5-fold increase (p < 0.001) in the number of apoptotic cells, addition of BKM120 was more effective showing a 4-fold increase, (p < 0.001) while GDC-0980 had no significant effect (Fig. 3B). As expected, the combination of BKM120/crizotinib resulted in more apoptotic cells than crizotinib alone (p < 0.001). The increase of apoptotic cells in the crizotinib/BKM120 group compared to BKM120 alone was not statistically significant (p > 0.05). The levels of cyclin D1 and cleaved–poly ADP-ribose polymerase (PARP) in the both MET and PI3K inhibitor treated cells were then determined via immunoblotting. Individual treatment of H2596 cells with BKM120 and GDC-0980 at 12 h did not have much effect on the levels of cleaved PARP, however the combination of crizotinib with BKM120 or GDC-0980 had much greater effect than single treatments (Fig. 3C). A significant decrease in cyclin D1 levels was observed in H2596 cells treated with any of the above three drugs at 24 h, with the most significant decrease observed in combination treated cells.

### Combination of MET and PI3K inhibitors inhibit downstream signaling pathways

**Figure 4:**
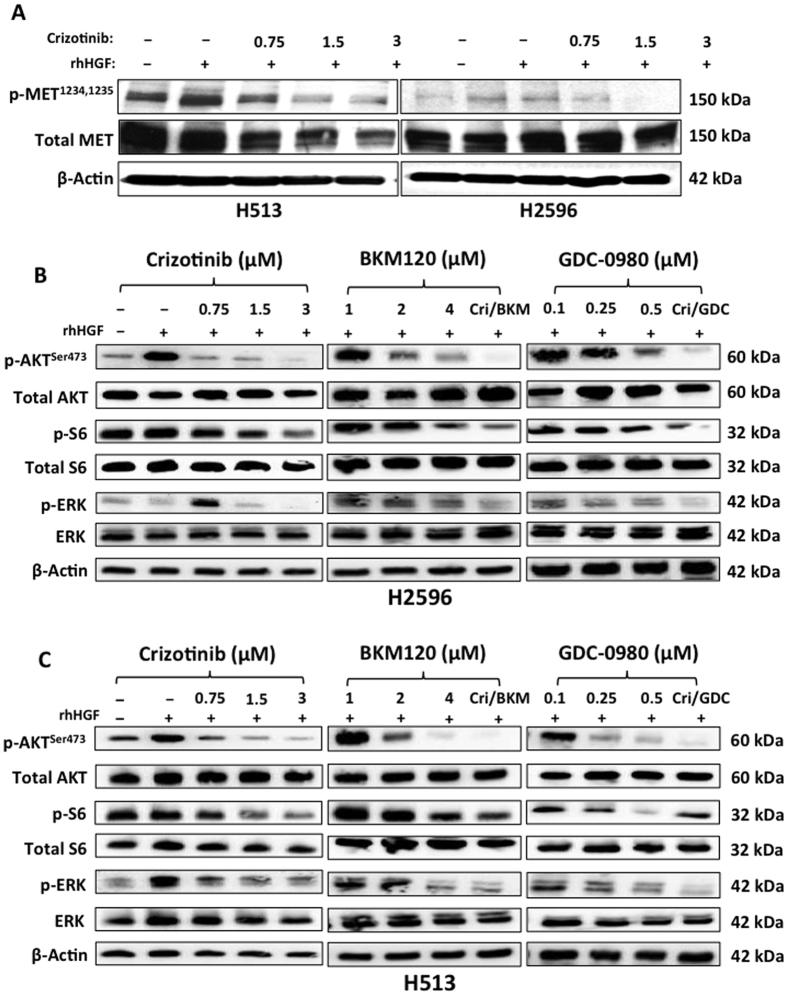
Effect of MET inhibitor crizotinib, BKM120 and GDC-0980 on downstream signaling pathway in mesothelioma cells. (**A**) Immunoblots of MPM cells treated with the indicated concentrations of crizotinib alone for 24 h with HGF stimulation. (**B**,**C**) Immunoblots of MPM cells treated with the indicated concentrations of crizotinib, BKM120 and GDC-0980, for 24 h.

In order to confirm that crizotinib is a MET tyrosine kinase inhibitor, H2596 and H513 cells were treated with increasing concentrations of crizotinib for 24 h followed by exposure to HGF and the whole cell lysates were then subjected to immunoblotting. As shown crizotinib indeed inhibited the phosphorylation MET, (active form) in both the cell lines (Fig. 4A). Next the combined effect of crizotinib with either BKM120 or GDC-0980 on downstream signaling was investigated. AKT phosphorylation was affected by both MET (crizotinib) and PI3K (BKM120 and GDC-0980) inhibitors in both H2596 and H513 cell lines. The effect was greater when a combination of MET and PI3K inhibitors was used. The activation of ribosomal S6 kinase, a downstream target of mTOR was inhibited by both BKM120 and GDC-0980 in both cell lines. The suppressive effect of crizotinib on phosphorylation of ribosomal S6 kinase was only seen at relatively higher concentration. Activation of MAPK, a known mediator of cell motility and metastasis, was completely suppressed by crizotinib alone and the combination of crizotinib and BKM120 in H513 cells (Fig. 4B,C).

To further confirm the effect on PI3K, the levels of PIP3 in MPM cells treated with both MET and PI3K inhibitors were determined by dot blot assay. Our results clearly show that treatment of H2596 cells with crizotinib had very little effect on the activity of PI3K, however BKM120 the class I PI3K inhibitor, significantly decreased PI3K activity. Treatment of H2596 cells with the combination of crizotinib and BKM120 further decreased PI3K activity as compared to single drug or vehicle treated cells. GDC-0980, the dual PI3K/mTOR inhibitor had no discernable effect on the level of PIP3, but the combination with crizotinib significantly decreased the activity of PI3K as compared to single drug or vehicle treated cells. A similar trend was observed with the H2373 cell line (Supplemental Fig. 2).

### MET and PI3K inhibitors inhibit anchorage-independent growth of mesothelioma cells

**Figure 5:**
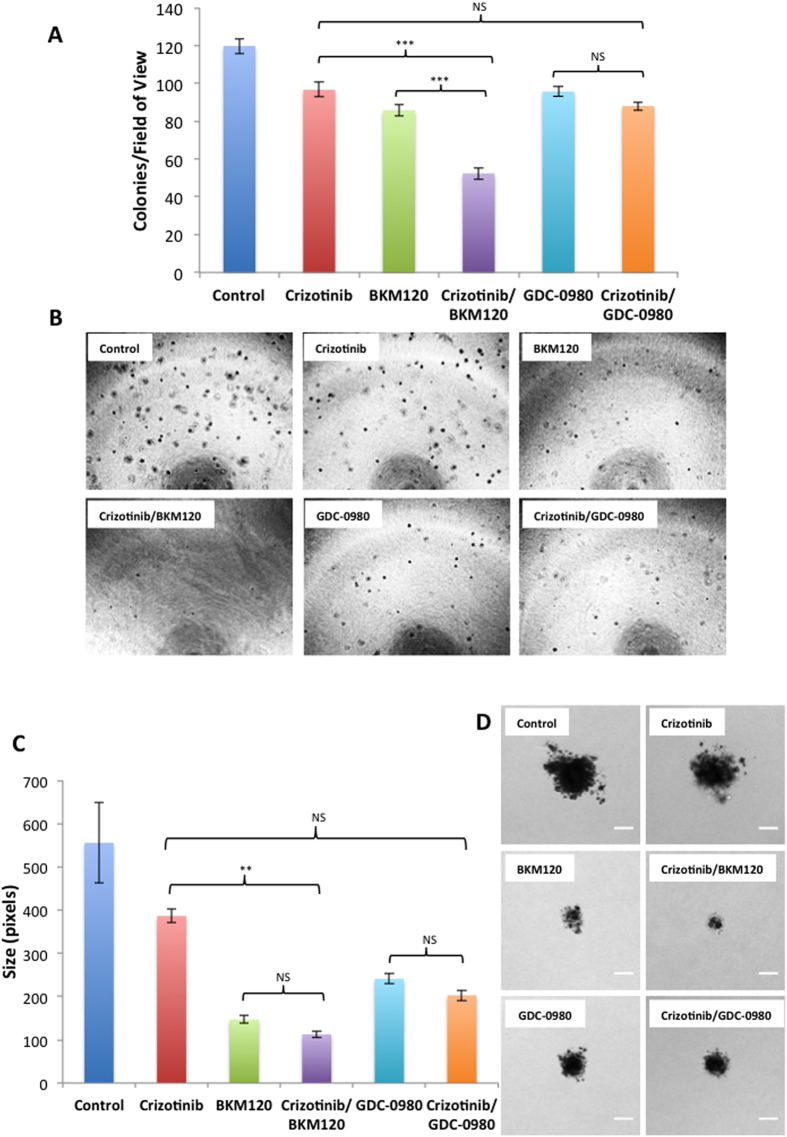
Crizotinib, BKM120 and GDC-0980 alone or in combination inhibit the anchorage independent growth of mesothelioma cells in a soft agar assay. H2596 cells were seeded in 0.4% Agarose and treated with 1 μM crizotinib, 2 μM BKM120, 0.25 μM GDC-0980 alone and in combination for a period of 30 days. Images of colonies were taken and colonies were counted using Image J. (**A**) Results are expressed as number of colonies per field of view ± SEM. Results were analyzed by ANOVA with Tukey post-test. (NS p > 0.05, *p < 0.05, **p < 0.01, ***p < 0.001) (BKM 120 Vs BKM120/crizotinib *p* < 0.001 GDC-0980 Vs GDC-0980/crizotinib *p* < 0.05). (**B**) Representative images of colonies. (**C**) Colony size in pixels for different groups ± SEM. (BKM120 Vs BKM120/crizotinib *p* < 0.01 GDC-0980 Vs GDC-0980/crizotinib *p* < 0.05) (**D**). Images of colonies were taken using a Zeiss Axiovert 200 m inverted optical microscope. Representative images of single colonies are shown at 10x magnification. Scale bar = 20 μm.

H2596 cells were plated in soft agar and then treated with either MET or PI3K inhibitors alone or in combination and to form colonies over 4 to 5 weeks. Our results show that for single drug treatment the cells treated with BKM120 formed the least number of colonies (p < 0.001) followed by those treated with GDC-0980 (p < 0.001) and crizotinib (p < 0.001) compared to vehicle treated controls. The combination of BKM120/crizotinib significantly decreased the number of colonies compared to either drug alone (p < 0.001). Combination of GDC 0980/crizotinib was not significantly more effective than either drug alone (p > 0.05) (Fig. 5A,B).

Along with total colony number we also determined colony size. Our results show the colonies were significantly smaller when cells were treated with BKM120 or GDC-0980 alone compared to the control (p < 0.001). The decrease observed with crizotinib was not statistically significant (p > 0.05). The exposure of cells to the combination of BKM120/crizotinib resulted in a further decrease in the size of the colonies compared to crizotinib treatment alone (p < 0.01). Combination of GDC-0980/crixotinib did not further reduce the colony sizes (p > 0.05) compared to either drug alone (Fig. 5C,D). The data demonstrates that dual inhibition of MET and PI3K decreases the capacity for colony formation and growth in MPM cells *in vitro*.

### BKM120 disrupts microtubule dynamics in mesothelioma cells

**Figure 6:**
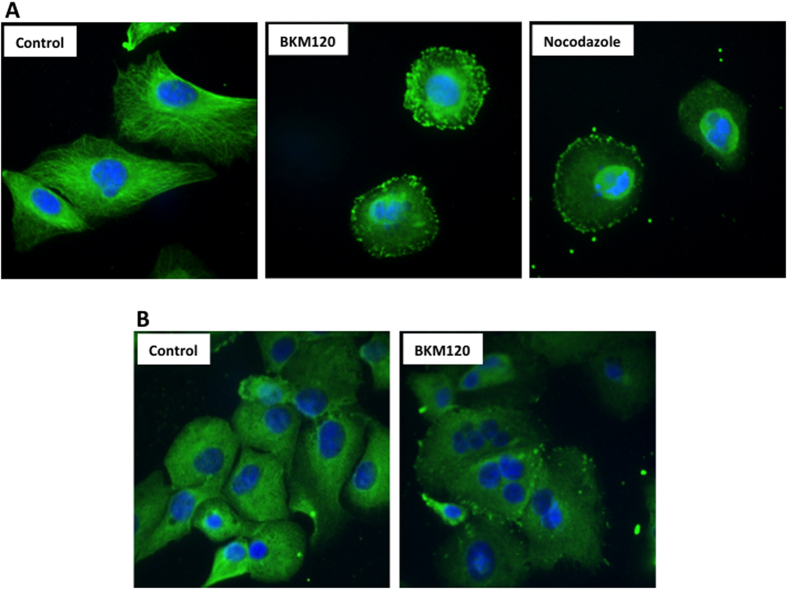
Effects of BKM120 on microtubule dynamics in mesothelioma cells. Images of H2596 cells treated with BKM120 (2 μM) and Nocodazole for 24 h and stained with DAPI (blue) and anti α-tubulin conjugated with FITC (green). (**B**) Images of H2596 cells treated with BKM120 for 72 h, showing mitotic catastrophe with multiple centrosomes.

Microtubules play a major role in maintaining cell morphology and in successful progression through the cell cycle. Given our earlier results demonstrating cell cycle arrest in BKM120 treated cells, we studied the effect of BKM120 on the dynamics of microtubules. Indirect immunofluorescence staining with an α-tubulin antibody of BKM120 treated H2596 cells show microtubule assembly in the cytoplasm is disorderly at 24 h with an increased number of multinucleated cells at 72 h (Fig. 6A,B). This microtubule misalignment increases the probability of a mitotic catastrophe and cell death. Conversely, microtubules in control cells formed an extensive organized network throughout the cytoplasm. Additionally, we used Nocodazole, an anti-neoplastic agent as a positive control, as it exerts its effects in cells by interfering with the polymerization of microtubules. The effect of BKM120 on MPM cells was comparable to Nocodazole as evidenced from disrupted microtubule polymerization (Fig. 6A,B).

### Combined use of MET and PI3K inhibitors shows anti tumor activity in a mouse PDX model

**Figure 7:**
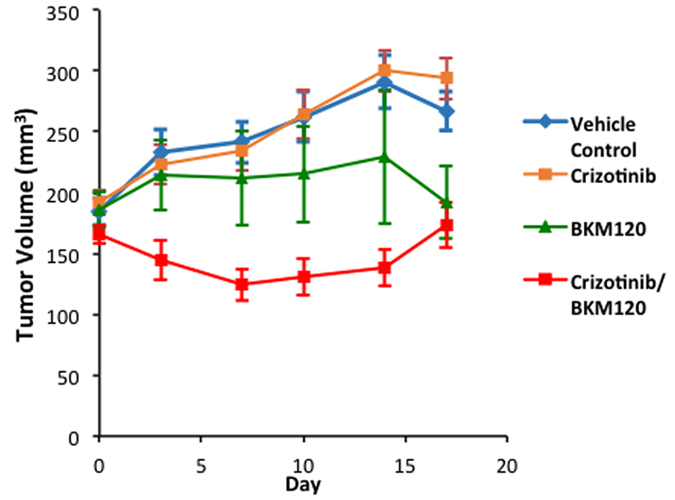
Combination of crizotinib and BKM120 synergistically inhibit the growth of patient derived xenografts in nude mice. Results from PDX experiment testing the efficacy of BKM120 and the combination of crizotinib/BKM120 in inhibiting the growth of tumors in nude mice. Female nude mice of age 5–8 weeks were implanted with low passage CTG-0234 human mesothelioma patient tumor. Oral gavage treatment with combination of crizotinib (25 mg/kg) and BKM120 (10 mg/kg) reduced PDX tumor growth significantly relative to vehicle control. The combined treatment was much more effective than BKM120 alone.

The antitumor activity of MET inhibitor crizotinib and pan-class I PI3K inhibitor BKM120 alone or in combination was further investigated in a mouse PDX mesothelioma model (conducted by Champions Oncology, Baltimore, MD). Female nude mice of age 5–8 weeks were implanted with a low passage CTG-0234 human mesothelioma patient tumor. At day 17 tumor volumes for the crizotinib and BKM120 groups were not significantly different compared to the vehicle alone treated control (DMSO/NMP-PEG300) group. Significantly lower tumor volumes were however observed in the crizotinib/BKM120 group (p ≤ 0.01). Analysis of data from Day 0 to Day 17 showed that the crizotinib/BKM120 combination group had significantly lower tumor volume compared to that of the vehicle group (p ≤ 0.0001) (Fig. 7). Despite no significant changes in body weight the combination treatment group exhibited increased morbidity and the experiment was concluded on day 17 (Supplemental Fig. 3).

## Discussion

MPM is a rare and invasive cancer that is generally associated with prior asbestos exposure. Although asbestos use is restricted in the US, the incidence of mesothelioma is still rising worldwide due to the relatively long latency period of the disease. Often chemotherapy and radiation therapy are ineffective and in general the prognosis is very poor. Hence, there is a pressing need for effective therapies.

Here we have studied the role of MET and it’s downstream signaling targets PI3K/mTOR in MPM and showed that a combinatorial approach to suppress both MET and PI3K activities is more effective than targeting individual pathways. In general combined treatment with MET inhibitor, crizotinib, and PI3K inhibitors, BKM120 or GDC-0980 resulted in a synergistic suppression of MPM cell growth (compare Fig. 1A–C). We also observed a similar effect of these inhibitors on the viability of peritoneal mesothelioma cells (Supplemental Fig. 4). The underlying mechanism of action appears to be cell cycle arrest leading to apoptosis. Once again the maximum apoptotic effect was observed when MPM cells were treated with both crizotinib and BKM120 (Fig. 3B,C). Cell motility, a reflection of metastatic potential was also decreased following treatment with crizotinib, BKM120 and GDC-0980 alone, with combination treatments being more effective (Fig. 2A,B). This was supported by the fact that the same combination of drugs had a very strong suppressive effect on HGF-induced MAPK activation (Fig. 4B,C). In addition, the phosphorylation of RTK downstream targets, AKT and S6 kinases, was also inhibited. Single treatment with crizotinib or BKM120 or GDC-0980 significantly reduced the number of colonies in a soft agar assay with the combination of crizotinib/BKM120 being even more effective (Fig. 5A). BKM120 and GDC-0980 alone also decreased the size of MPM colonies in soft agar and combination of crizotinib/BKM120 had the most significant effect (Fig. 5C). These results suggest that the combination of MET, PI3K and mTOR inhibition is the most effective in decreasing the transformation potential of MPM cells. Finally, using a mesothelioma PDX mouse model, we demonstrated that the combination of crizotinib/BKM120 was more effective than the single drugs alone in inhibiting MPM tumor growth *in vivo* (Fig. 7).

Crizotinib, the focus of this paper, was originally developed as a MET inhibitor, and was later shown to also be a potent inhibitor of ALK. Of the 120 kinases originally tested, crizotinib was found to selectively inhibit ALK and MET kinases^20^. Studies have shown that it significantly inhibits MET phosphorylation and signal transduction, tumor cell proliferation and could induce apoptosis in breast and gastric cancer cells. The antitumor activity of crizotinib is mediated by its ability to inhibit MET activity that supports both tumor growth, and metastasis. In this study, we did confirm the ability of crizotinib to inhibit HGF induced MET tyrosine kinase activity at the concentrations used (Fig. 4A). MAPK, another downstream target of MET and major player in metastasis was also more strongly inhibited by the combination treatments compared to single drug treatments^21^. In addition, BKM120 and also GDC-0980 were effective in decreasing phosphorylation of AKT and S6 ribosomal kinases which are downstream targets of PI3K. These results concur with previously published reports^22^,^23^,^24^. The underlying reason could be the fact that PI3K is a key downstream signal transducer for RTKs including MET. BKM120 is known to significantly suppress cell proliferation by decreasing the levels of p-AKT and its action is synergistic when combined with other targeted agents such as MEK or HER2 inhibitors or with cytotoxic agents such as docetaxel or temozolomide in multiple cancer cell lines^25^. Our results combining crizotinib and BKM120 align with the above findings.

Our results show that MET inhibitor (crizotinib) and pan-class I PI3K inhibitor (BKM120) when used alone significantly suppressed the viability of MPM cells in a dose dependent manner. This is in agreement with previous studies carried out with these inhibitors in other cancers^20^,^22^,^23^,^26^,^27^,^28^,^29^,^30^,^31^,^32^,^33^,^34^,^35^,^36^. In addition, dual targeting of MET and PI3K is highly synergistic in suppressing cell viability thereby paving the way for a combinatorial approach in treating MPM. This builds on our previous finding showing synergistic suppressive activity of MET inhibitor ARQ-197 with GDC-0980 in MPM^19^.

Cell cycle analysis results showed that treatment with crizotinib and GDC-0980 mostly arrested MPM cells at G0-G1 phase, whereas BKM120 induced cell cycle arrest at G2-M phase. However the combination with crizotinib arrested cells in G2-M phase (Fig. 3A). Also our studies revealed that both MET and PI3K inhibitors induced apoptosis in MPM cells, while maximum effect was seen with BKM120 (Fig. 3B). As arrest in G2-M phase could trigger cell apoptosis, this could be the underlying mechanism triggering apoptosis seen in the present study. Moreover treatment of MPM cells with both MET and PI3K inhibitors significantly decreased the levels of cyclin-D1, the cell cycle regulator and increased the levels of cleaved-poly (ADP-ribose) polymerase (PARP), a marker of apoptosis (Fig. 3C). Both crizotinib and BKM120 are known to induce apoptosis in various cancer cell lines^22^,^26^,^31^. Ren H *et al*. (2013) have demonstrated that BKM120 enhanced TRAIL-induced apoptosis in lung cancer^29^. Another study in hepatocellular carcinoma showed that BKM120 markedly reduced tumor growth mainly via cell cycle arrest than by apoptosis^26^. BKM120 also effectively suppressed neuroendocrine tumor cell proliferation and stimulated apoptosis^24^. Here, we have demonstrated that the combination of crizotinib/BKM120 induces apoptosis in MPM cells via G2-M cell cycle arrest.

In addition we have shown that treatment of MPM cells with BKM120 resulted in disruption of microtubule assembly causing mitotic catastrophe cell death (Fig. 6A,B). This is in agreement with previous reports on BKM120 induced disruption of microtubule equilibrium in multiple cancer cell lines^37^. Treatment of glioma cells with BKM120 inhibited microtubule dynamics and induced either robust G2-M arrest and apoptosis or mitotic catastrophe^33^.

Finally, using a PDX model we have demonstrated that the combination of crizotinib/ BKM120 is effective in suppressing MPM tumor growth. This result is the first of its kind, although others have attempted similar approaches. For instance treatment with BKM120 significantly inhibited tumor growth *in vivo* and it also showed synergistic cytotoxicity with dexamethasone in dexamethasone sensitive multiple myeloma cells^31^. Moreover, combined treatment of neuroendocrine tumors with BKM120 and MEK inhibitor (PD0325901) was more effective in suppressing tumor growth in a xenograft model^24^. Recently a combination of PI3K/MEK inhibition with BKM120 and PD0325901 was effective in inducing tumor regression in mouse model of cancer that harbored a *KRAS* mutation. It is interesting to note that MEK was a key downstream target in the MET signaling pathway and active MEK cascade was implicated in tumor cell motility and metastasis^27^. There are several recent studies that support the use of crizotinib to target MET in suppressing tumor growth. Crizotinib alone strongly inhibited gastric carcinoma tumor growth in a *MET* amplified PDX model^38^. In contrast the MPM tissue used to generate the PDX model in this study was not MET amplified. Despite this, the combination of crizotinib/BKM120 did suppress MPM tumor growth. Recently it was also shown that in papillary thyroid carcinoma crizotinib acts as a antitumor agent by inhibiting phosphorylation of MET, AKT and its downstream signaling molecules and synergizes with TRAIL in suppression of tumor growth^22^. Surriga and coworkers have shown that inhibition of MET activity by crizotinib was sufficient to strongly suppress metastasis of uveal melanoma^36^.

BKM120 is a pan inhibitor of all four class I PI3K isoforms and inhibited tumor cells bearing PIK3CA mutations. Previously using a panel of 353 cell lines, there was dose dependent decrease in PI3K activity *in vivo* as measured by inhibition of phosphorylation of AKT. It also suppressed tumor growth in multiple xenograft mouse models and its action was synergistic when combined with MEK or HER2 inhibitors^25^. Our *in vivo* results are very similar to the above (Fig. 7).

In conclusion, our results demonstrate the potential of a dual MET/PI3K targeting strategy in treating MPM. While individual targeting of each kinase may show modest suppressive activity, the combination of crizotinib/BKM120 demonstrated superior efficacy in inhibiting multiple aspects of tumor cell growth *in vitro* (viability, migration, colony formation) and *in vivo* (PDX). Patients with MPM will likely benefit from combination treatments as has been demonstrated for other cancer types.

## Methods

### Antibodies

AKT, p-AKT^ser473^, S6, p-S6^Ser235/236^, cyclin D1, cleaved PARP, p-MET (1234/1235), and anti-MAPK antibodies (ERK and p-ERK) were from Cell Signaling (Danvers, MA, USA). β-actin and α-tubulin antibody were from Sigma (St. Louis, MO, USA). PIP3 antibody was from MBL Co. Ltd (Japan). MET and Alexa-Fluor 488 (mouse) antibodies were from Molecular Probes (Grand Island, NY, USA).

### Cell lines

Mesothelioma cell lines namely H2596, H513, H2461, H2052, H2452, H28 and H2373 and one benign transformed mesothelial control cell line Met-5A, were obtained from American Type Culture Collection (ATCC) (Manassas, VA, USA). Peritoneal mesothelioma cell lines, HAY, ROB and YOU were kind gift from Dr. Raffit Hassan (National Cancer Institute, Bethesda, MD). All the cell lines except for Met-5A were cultured in RPMI 1640 medium (Gibco/BRL) supplemented with 10% (v/v) fetal bovine serum (FBS), L-glutamine and 1% penicillin-streptomycin at 37 °C with 5% CO_2_. Met-5A cells were cultured in M199 media according to manufactures instructions (ATCC).

### Small molecule inhibitors and other reagents

Recombinant human HGF was purchased from R&D Systems (Minneapolis, MN, USA) GDC-0980 was kind gift from Genentech (San Francisco, CA, USA), crizotinib and BKM120 were purchased from Selleck Chemicals (Houston, TX, USA). Stock solutions were prepared in DMSO and stored at −20 °C.

### Immunoblotting

Cells were treated with the indicated concentrations of inhibitors for the given time. Whole cell lysates were prepared using RIPA lysis buffer and proteins were detected by immunobloting as previously described^39^.

### Viability Assays

Exponentially growing cells were plated overnight in 96 well flat bottom plates and treated with the indicated drugs, for 72 h. Cell viability was measured using Alamar Blue method as described previously^40^. Each experiment was repeated at least three times. IC_50_ values were generated for all the cell lines using GraphPad Prism software.

### Synergy Assays

The cells were treated with either a single drug or with a combination of two drugs for 72 h. The synergy assays were done as previously described^19^. The analysis of synergy assay was done by the isobologram and combination-index methods, derived from the median-effect principle of Chou and Talalay using ‘Compusyn’ software^41^.

### Cell Cycle Analysis

The cells were treated with the indicated drugs for 48 h, then washed, harvested, fixed in 70% ethanol and cell cycle analysis was done using propidium iodide as previously described^19^. Samples were analyzed by LSRII flow cytometer (BD Bioscience San Jose, CA, USA). The percentage of cells in different phases of the cell cycle was calculated using FlowJo 9.3.0 software (Tree Star Inc., Ashland, OR, USA).

### Apoptosis Assay

The cells were treated with the indicated drugs and their combinations for 48 h. Apoptosis was evaluated using the Annexin V apoptosis kit from BD Biosciences (San Jose, CA, USA) as per the manufacture’s protocol as previously described^19^.

### Dot Blot Assay to estimate PIP3 activity

The assay was carried out as previously described^42^. In brief, the cell lysates containing 50 μg of protein was spotted directly onto a nitrocellulose membrane. After blocking of the membrane with 5% BSA in 0.05% Tween/TBS for an hour, it was incubated with PIP3 antibody overnight at 4 °C. The next day the membrane was washed and incubated with secondary antibody and proteins were visualized using an enhanced chemiluminescence reagent. Densitometric analysis was done using ImageJ software (NIH, Bethesda, MA).

### Soft Agar colony formation Assay

Soft agar assay for colony formation was done as previously described^43^. Briefly, a base agar layer (Invitrogen) and top agar layer, containing 2.5 × 10^3^ cells per well, were plated in 24-well tissue culture dishes (Ibidi, Madison, Wisconsin, USA). Cells were grown for 4 to 5 weeks at 37 °C in a humidified atmosphere containing 5% CO_2_. Viable colonies were photographed with a Zeiss Axiovert 200 M with a Hammatsu Orca ER camera and counted using ImageJ software and custom written macros.

### Immunofluorescence and Confocal Microscopy

Cells were grown in 10% FBS media on glass coverslips in 6-well tissue culture plates overnight. The next day they were treated with the indicated concentrations of BKM120 for 24 h and immunofluroscence staining was carried out, as described previously^44^. In brief the cells were then washed with PBS and then fixed with 1.5% glutaraldehyde for 10 min at RT and then permeabilized with 1% Triton X-100 in PBS for 60 min. The cells were then blocked in 1% sodium borohydride 3 times for 10 min. The cells were then stained with α-tubulin ((Sigma, St Louis, MO, USA) and then with secondary antibody Alexa-Fluor 488 for 30 min at 37 °C. After washing with PBS the coverslips were mounted on slides using Vectashield. The fluorescent pictures were visualized and captured using Olympus DSU spinning disk confocal microscope equipped with CCD camera. Nocodazole (0.4 μg/ml) was used as a positive control^45^.

### Cell Migration Assay

H2596 cells were plated (2.5 × 10^5^) in 60 mm plates overnight. Next day the cells were treated with crizotinib, GDC-0980 and BKM120 alone or in combination for 16 h. After the treatment, the cells were trypsinized, counted and replated for migration assay into Transwell chambers (BD Biosciences, San Jose, CA) containing 500 μl serum free media with indicated inhibitors at a density of 2.5 × 10^4^ cells per chamber. The chambers were placed into a 24-well plate containing 700 μl RPMI with 10% FBS as a chemo attractant. After 7 h, the top and bottom chambers were washed twice with PBS. The cells were then fixed in 4% paraformaldehyde for 10 min at room temperature and washed again with PBS. After removing non-migrated cells from the top chamber using a cotton swab, the remaining cells were stained with Crystal violet for 30 minutes and then rinsed thoroughly with distilled water to remove the extra stain. Images of each chamber were captured Zeiss Axiovert 200 M with a Hammatsu Orca ER camera and the migrated cells were counted using ImageJ and a custom written macro.

### Patient Derived mouse xenograft model

CTG-0234 is a low passage TumorGraft^®^TM model of Human Mesothelioma with two fold increase of MET gene copy number in Immunocompromised Mice (PDX) established at Champions Oncology (Baltimore, MD). The patient tumor tissue was implanted subcutaneously into 5–8 week old female mice (Taconic, NCr nude) according to standard protocol. Affymetrix expression data of the xenografted tumor from Champions Oncology is included in Supplementary Table 1. Once the tumor volume was reached to an average of 200 mm^3^, mice were randomized and distributed into four groups. Mice were treated daily by oral gavage with vehicle, crizotinib (25 mg/kg/dose), BKM120 (10 mg/kg/dose) or combination. After initiation of the treatment, the mice were weighed and tumor volumes measured twice a week.

### Ethics Statement

For mouse models of mesothelioma, all animal experimental procedures were approved by the University of Chicago’s Institutional Animal Care and Use Committee (IACUC). All experimental methods were performed in accordance with the approved guidelines. All efforts were made to minimize animal suffering.

### Statistical Analysis

Statistical analysis was performed using GraphPad Prism version 5.0 (GraphPad Inc, San Diego, CA). One-way ANOVA with Tukey multiple comparison post-test used when appropriate. For the PDX mouse experiment one-way ANOVA followed by Dunnett’s multiple comparison test comparing vehicle Vs. treatment was used.

## Additional Information

**How to cite this article**: Kanteti, R. *et al*. PI3 Kinase Pathway and MET Inhibition is Efficacious in Malignant Pleural Mesothelioma. *Sci. Rep.* **6**, 32992; doi: 10.1038/srep32992 (2016).

## Supplementary Material

Supplementary Information

Supplementary Dataset 1

Supplementary Dataset 2

Supplementary Dataset 3

Supplementary Dataset 4
