# Supplementary material for: PI3 Kinase Pathway and MET Inhibition is Efficacious in Malignant Pleural Mesothelioma
Source: Sci Rep. 2016 Sep 13;6:32992. doi: 10.1038/srep32992 (PMC5021085; doi:10.1038/srep32992)
Supplement: Supplementary Information [file srep32992-s1.pdf]

**PI3 Kinase Pathway and MET Inhibition is Efficacious in Malignant Pleural Mesothelioma**

Rajani Kanteti[1], Jacob J. Riehm[1], Immanuel Dhanasingh[1], Frances E. Lennon[1], Hedy L. Kindler[1], Ravi Salgia[1*]

**Supplementary Figure legends:**

**Supplemental Figure 1: (A) Combination Index plot and Isobologram for combination of Crizotinib and BKM120 in H513 cells.** The left side panel shows the CI plot for the combinations of drugs where synergy (identified by a Combination Index <1) over a range of drug concentrations. The red square in the isobologram represents concentrations of both drugs that inhibit cellular proliferation by 75% (Fraction affected = 0.75). A combination index (CI) value of 0.07 was calculated using CompuSyn software. The line represents an additive affect, where CI = 1. **(B) Combination Index plot and Isobologram for combination of Crizotinib and GDC-0980 in H2052 cells.** The left side panel shows the CI plot where synergy (identified by CI<1) is demonstrated over a range of drug combinations. The green triangle in the isobologram represents concentrations of both drugs that inhibit cellular proliferation by 90% (Fraction affected = 0.9). A combination index (CI) value of 0.49 was calculated using CompuSyn software. The line represents an additive affect, where CI = 1.

**Supplemental Figure 2: Effect of crizotinib, BKM120 and GDC-0980 on PIP3 levels of mesothelioma cells.** Dot blot assay showing PIP3 levels in the H2596 and H2373 cells treated with MET and PI3K inhibitors and densitometric anlaysis of the blot.

**Supplemental Figure 3: Effect of combined treatment of crizotinib and BKM-120 on mouse body weight during PDX model.** Female nude mice of age 5-8 weeks were implanted with low passage CTG-0234 human mesothelioma patient tumor. Mice were then treated daily by oral gavage with vehicle, crizotinib (25 mg/kg), BKM120 (10mg/kg) and their combination. Body weights were recorded twice a week.

**Supplemental Figure 4: Effect of crizotinib, BKM120 and GDC-0980 on viability of peritoneal mesothelioma cells.** Peritoneal Mesothelioma cell lines ROB, HAY, and YOU were treated with crizotinib, BKM120 and GDC-0980 for 72 h. Viability was measured by Alamar Blue assay. The data shown represents the average ± SEM.

**Supplemental Table 1:** Affymetrix expression data of the xenografted tumor (CTG-0234 low passage TumorGraft model Human Mesothelioma) from Champions Oncology.

Kanteti, *et al.* Supplementary Figure 1

A

## Crizotinib and BKM120 in H513

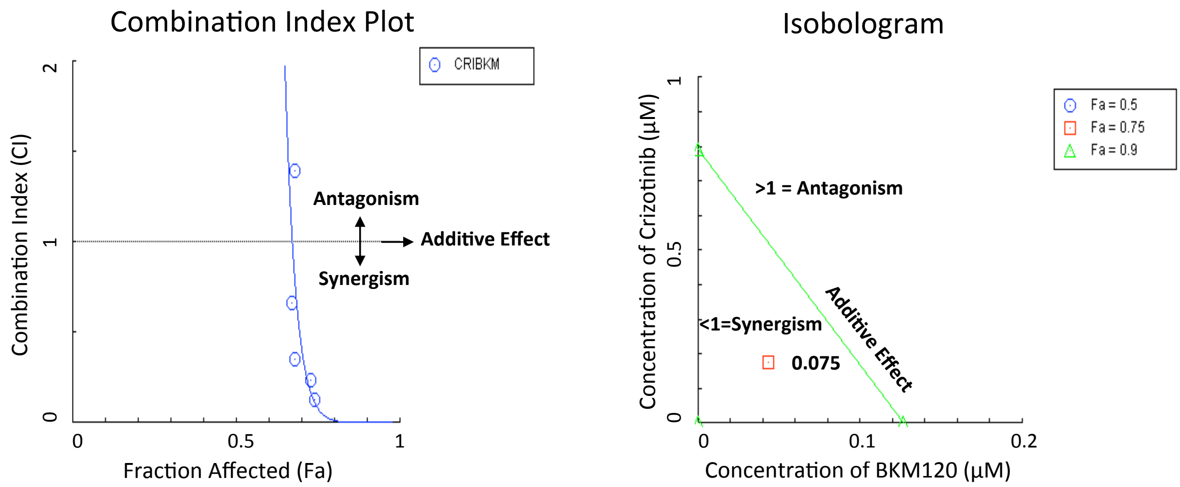


B

## Crizotinib and GDC-0980 in H2052

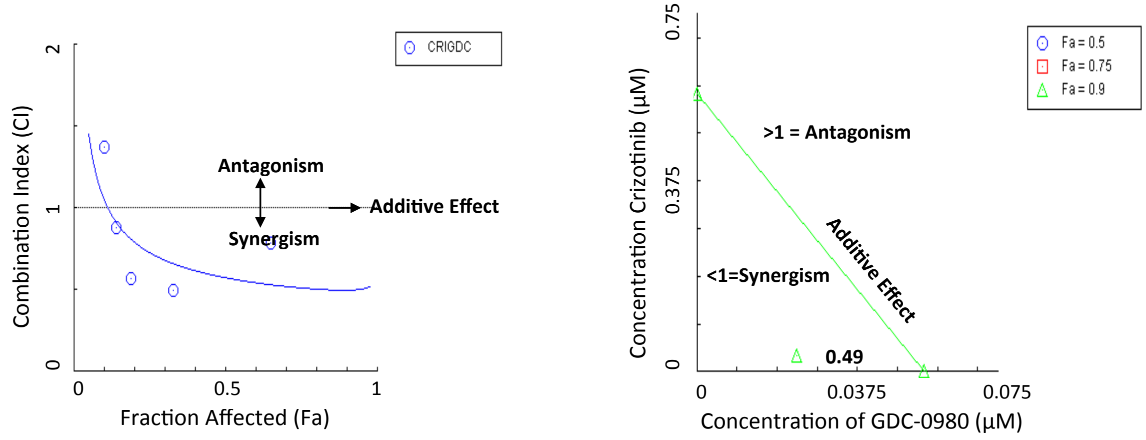

Kanteti, *et al.* Supplementary Figure 2

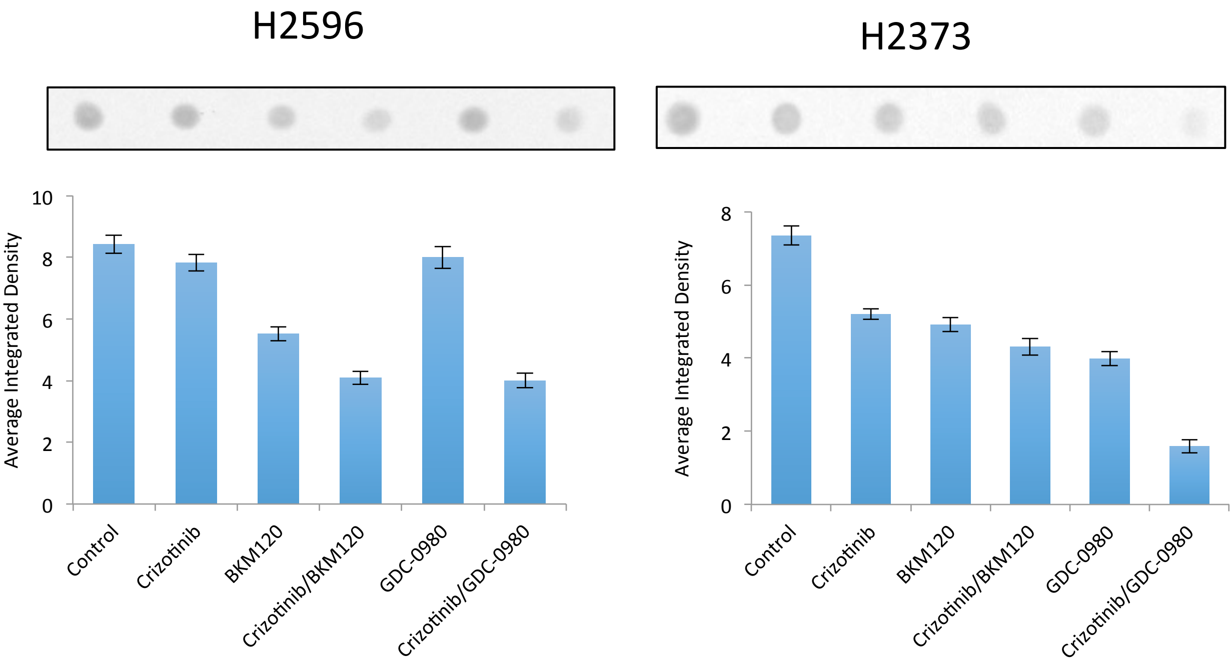

Kanteti, *et al.* Supplementary Figure 3

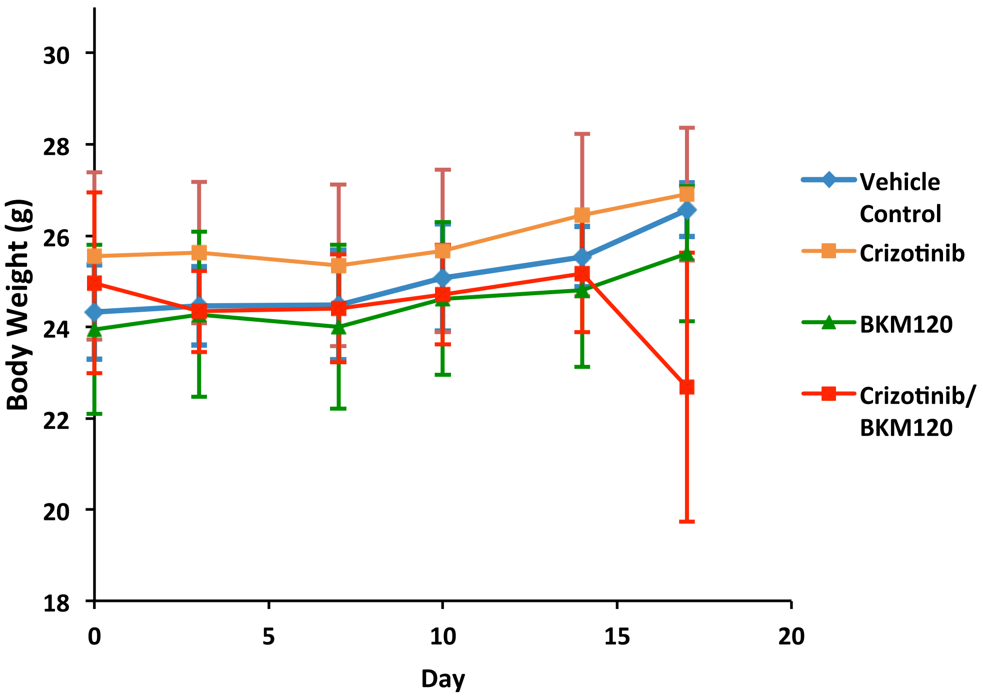

Kanteti, *et al.* Supplementary Figure 4

**A**

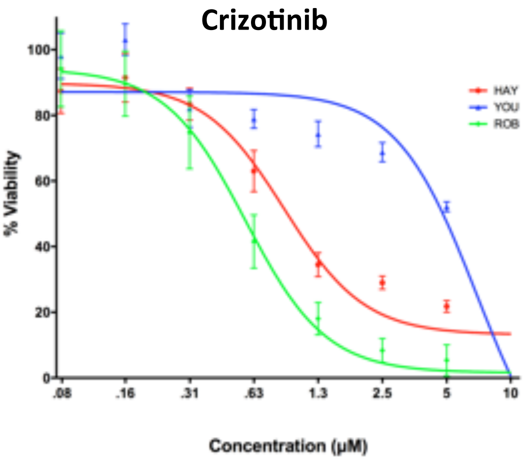


**B**

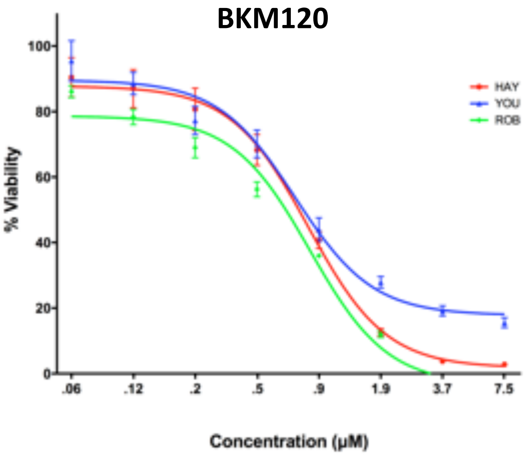


**C**

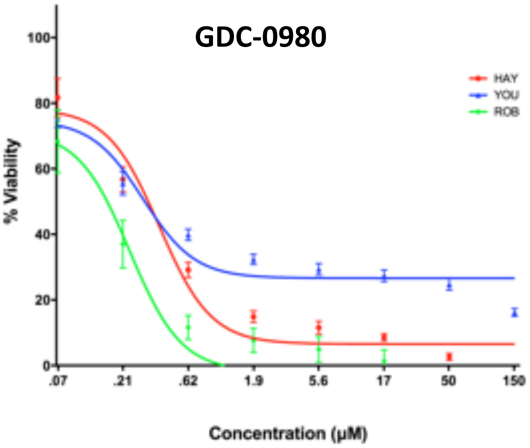

Kanteti, *et al.* Supplementary Table 1

Affymetrix Gene Expression of CTG-0234 TumorGraft model of Mesothelioma

| Model | Cancer type | Gene | Fold change | Over or Under Mean | Adjusted p-value | Array |
|---|---|---|---|---|---|---|
| CTG-0234 | Mesothelioma | *MTOR* | 0.67 | Under | 1.31E-35 | hgu219 |
| | | *MET* | 2.26 | Over | 4.83E-24 | hgu219 |
| | | *NF2* | 1.25 | Over | 1.71E-29 | hgu219 |
| | | *BAP1* | 0.68 | Under | 1.47E-81 | hgu219 |
| | | *AKT1* | 0.92 | Under | 1.78E-07 | hgu219 |
| | | *AKT2* | 0.83 | Under | 1.46E-10 | hgu219 |
| | | *AKT3* | 8.53 | Over | 6.21E-83 | hgu219 |
| | | *PIK3CA* | 0.87 | Under | 2.08E-07 | hgu219 |
| | | *CUX1* | 1.33 | Over | 7.52E-29 | hgu219 |
| | | *PTK2* | 0.50 | Under | 6.24E-60 | hgu219 |
| | | *MST1R* | 0.86 | Under | 7.81E-15 | hgu219 |
| | | *PXN* | 1.31 | Over | 3.12E-07 | hgu219 |
