# Supplementary material for: PI3 Kinase Pathway and MET Inhibition is Efficacious in Malignant Pleural Mesothelioma
Source: Sci Rep. 2016 Sep 13;6:32992. doi: 10.1038/srep32992 (PMC5021085; doi:10.1038/srep32992)
Supplement: Supplementary Dataset 1 [file srep32992-s2.pdf]

CompuSyn Report

**Experiment Name:**	H2052 Plate 1&2 average
**Date:**	9-9-13
**File Name:**	C:\Documents and Settings\idhanasingh\Desktop\CRIZOTINIB-GDC SYNERGY\9-9-13 STEP-2\H2052 AVERGE OF PLATE1&2.cse
**Description**	Synergy step-2 Average of plate 1 and 2 for H2052

**Drug:**	Crizotinib (Cri) [uM]
**Drug:**	GDC0980 (GDC) [uM]
**Drug Combo:**	CRIZO-GDC (CRIGDC) (Cri+GDC [1:0.75])

Data for Drug: Cri [uM]

Dose	Effect
4.0	0.18
2.0	0.32
1.0	0.51
0.5	0.93
0.25	0.99

5 data points entered.

**X-int:**	0.19155
**Y-int:**	0.43010 +/- 0.13958
**m:**	-2.2453 +/- 0.32786
**Dm:**	1.55437
**r:**	-0.9695

Data for Drug: GDC [uM]

Dose	Effect
3.0	0.24
1.5	0.31
0.75	0.39
0.375	0.59
0.188	0.8

5 data points entered.

**X-int:**	-0.1873
**Y-int:**	-0.1688 +/- 0.05769
**m:**	-0.9011 +/- 0.13015

**Dm:**	0.64961
**r:**	-0.9701

Data for Drug Combo: CRIGDC (Cri+GDC [1:0.75])

Dose A	Effect
4.0+	0.1
2.0+	0.14
1.0+	0.19
0.5+	0.33
0.25+	0.65

5 data points entered.

**X-int:**	-0.2529
**Y-int:**	-0.2459 +/- 0.08206
**m:**	-0.9723 +/- 0.16739
**Dm:**	0.55860
**r:**	-0.9583

Dose-Effect Curve

Median-Effect Plot

CI Data for Drug Combo: CRIGDC (Cri+GDC [1:0.75])

Fa	CI Value	Total Dose
0.05	1.43331	11.5412
0.1	1.04770	5.35186
0.15	0.88469	3.32561
0.2	0.79010	2.32433
0.25	0.72672	1.72903
0.3	0.68056	1.33522
0.35	0.64506	1.05584
0.4	0.61671	0.84763
0.45	0.59341	0.68664
0.5	0.57389	0.55860
0.55	0.55727	0.45443
0.6	0.54300	0.36813
0.65	0.53069	0.29553

Fa	CI Value	Total Dose
0.7	0.52011	0.23369
0.75	0.51119	0.18047
0.8	0.50401	0.13425
0.85	0.49904	0.09383
0.9	0.49765	0.05830
0.95	0.50512	0.02704
0.97	0.51597	0.01565

CI values for actual experimental points:

Total Dose	Fa	CI Value
7.0	0.1	1.37035
3.5	0.14	0.88125
1.75	0.19	0.56824
0.875	0.33	0.49772
0.4375	0.65	0.78563

Combination Index Plot

Logarithmic Combination Index Plot

DRI Data for Drug Combo: CRIGDC (Cri+GDC [1:0.75])

Fa	Dose Cri	Dose GDC	DRI Cri	DRI GDC
0.05	5.76862	17.0525	0.87470	3.44756
0.1	4.13565	7.44134	1.35231	3.24432
0.15	3.36560	4.45328	1.77105	3.12454
0.2	2.88199	3.02556	2.16987	3.03728
0.25	2.53541	2.19862	2.56616	2.96705
0.3	2.26693	1.66351	2.97116	2.90703
0.35	2.04781	1.29125	3.39413	2.85357
0.4	1.86200	1.01876	3.84426	2.80444
0.45	1.69968	0.81165	4.33186	2.75812
0.5	1.55437	0.64961	4.86957	2.71348
0.55	1.42147	0.51992	5.47404	2.66956
0.6	1.29756	0.41422	6.16835	2.62547
0.65	1.17982	0.32681	6.98640	2.58027
0.7	1.06578	0.25367	7.98098	2.53282

Fa	Dose Cri	Dose GDC	DRI Cri	DRI GDC
0.75	0.95292	0.19193	9.24055	2.48158
0.8	0.83833	0.13947	10.9282	2.42420
0.85	0.71787	0.09476	13.3891	2.35651
0.9	0.58420	0.05671	17.5349	2.26950
0.95	0.41883	0.02475	27.1097	2.13571
0.97	0.33052	0.01372	36.9623	2.04534

DRI values calculated at experimental points

Fa	Dose Cri	Dose GDC	DRI Cri	DRI GDC
0.1	4.13565	7.44134	1.03391	2.48045
0.14	3.48875	4.87045	1.74437	3.24697
0.19	2.96495	3.24724	2.96495	4.32966
0.33	2.13075	1.42553	4.26149	3.80141
0.65	1.17982	0.32681	4.71930	1.74297

DRI Plot for Combo: CRIGDC (Cri+GDC [1:0.75])

Log(DRI) Plot for Combo: CRIGDC (Cri+GDC [1:0.75])

Isobologram for Combo: CRIGDC (Cri+GDC [1:0.75])

Polygonogram at Fa = 0.9

Summary Table

**Experiment Name:**	H2052 Plate 1&2 average
**Date:**	9-9-13
**File Name:**	C:\Documents and Settings\idhanasingh\Desktop\CRIZOTINIB-GDC SYNERGY\9-9-13 STEP-2\H2052 AVERGE OF PLATE1&2.cse
**Description**	Synergy step-2 Average of plate 1 and 2 for H2052

**Drug:**	Crizotinib (Cri) [uM]
**Drug:**	GDC0980 (GDC) [uM]
**Drug Combo:**	CRIZO-GDC (CRIGDC) (Cri+GDC [1:0.75])

Drug/Combo	Dm	m	r
Cri	1.55437	-2.2453	-0.9695
GDC	0.64961	-0.9011	-0.9701
CRIGDC	0.55860	-0.9723	-0.9583

CI values at:

Combo	ED50	ED75	ED90	ED95
CRIGDC	0.57389	0.51119	0.49765	0.50512

Data for Fa = 0.5

Drug/Combo	CI value	Dose Cri	Dose GDC
Cri		1.55437	
GDC			0.64961
CRIGDC	0.57389	0.31920	0.23940

Data for Fa = 0.75

Drug/Combo	CI value	Dose Cri	Dose GDC
Cri		0.95292	
GDC			0.19193
CRIGDC	0.51119	0.10312	0.07734

Data for Fa = 0.9

Drug/Combo	CI value	Dose Cri	Dose GDC
Cri		0.58420	
GDC			0.05671
CRIGDC	0.49765	0.03332	0.02499

Data for Fa = 0.95

Drug/Combo	CI value	Dose Cri	Dose GDC
Cri		0.41883	
GDC			0.02475
CRIGDC	0.50512	0.01545	0.01159

Data for Fa = 0.97

Drug/Combo	CI value	Dose Cri	Dose GDC
Cri		0.33052	
GDC			0.01372
CRIGDC	0.51597	0.00894	0.00671
