# Supplementary material for: PI3 Kinase Pathway and MET Inhibition is Efficacious in Malignant Pleural Mesothelioma
Source: Sci Rep. 2016 Sep 13;6:32992. doi: 10.1038/srep32992 (PMC5021085; doi:10.1038/srep32992)
Supplement: Supplementary Dataset 2 [file srep32992-s3.pdf]

CompuSyn Report

**Experiment Name:**	H2596 PLATE-1
**Date:**	10-28-13
**File Name:**	C:\Documents and Settings\idhanasingh\Desktop\Crizotinib BKM synergy\STEP-2 10-28-13\H2596 PLATE-1.cse
**Description**	SYNERGY STEP-2 BETWEEN CRI AND BKM

**Drug:**	CRIZOTINIB (CRI) [uM]
**Drug:**	BKM120 (BKM) [uM]
**Drug Combo:**	CRI-BKM120 (CRIBKM) (CRI+BKM [1:2])

Data for Drug: CRI [uM]

Dose	Effect
2.5	0.62
1.25	0.76
0.63	0.84
0.31	0.97
0.16	0.99

5 data points entered.

**X-int:**	0.44411
**Y-int:**	0.67886 +/- 0.09245
**m:**	-1.5286 +/- 0.19709
**Dm:**	2.78040
**r:**	-0.9760

Data for Drug: BKM [uM]

Dose	Effect
5.0	0.65
2.5	0.7
1.25	0.75
0.625	0.93
0.313	0.98

5 data points entered.

**X-int:**	0.75400
**Y-int:**	0.90154 +/- 0.11068
**m:**	-1.1957 +/- 0.25359

Dm: 5.67549
r: -0.9387

Data for Drug Combo: CRIBKM (CRI+BKM [1:2])

Dose A	Effect
2.5+	0.57
1.25+	0.59
0.63000+	0.64
0.31+	0.71
0.16000+	0.82

5 data points entered.

X-int: 1.00148
Y-int: 0.43503 +/- 0.04394
m: -0.4344 +/- 0.08705
Dm: 10.0342
r: -0.9447

Dose-Effect Curve

Median-Effect Plot

CI Data for Drug Combo: CRIBKM (CRI+BKM [1:2])

Fa	CI Value	Total Dose
0.05	242.238	8816.23
0.1	74.4666	1578.45
0.15	35.9545	544.135
0.2	20.8054	244.048
0.25	13.2517	125.851
0.3	8.94079	70.5660
0.35	6.25681	41.7240
0.4	4.48200	25.5187
0.45	3.25613	15.9261
0.5	2.38161	10.0342
0.55	1.74255	6.32199
0.6	1.26725	3.94551
0.65	0.90943	2.41310

Fa	CI Value	Total Dose
0.7	0.63819	1.42681
0.75	0.43233	0.80003
0.8	0.27699	0.41256
0.85	0.16173	0.18504
0.9	0.07926	0.06379
0.95	0.02514	0.01142
0.97	0.01113	0.00336

CI values for actual experimental points:

Total Dose	Fa	CI Value
7.5	0.57	2.19638
3.75	0.59	1.16766
1.89	0.64	0.68936
0.93	0.71	0.43128
0.48	0.82	0.35559

Combination Index Plot

Logarithmic Combination Index Plot

DRI Data for Drug Combo: CRIBKM (CRI+BKM [1:2])

Fa	Dose CRI	Dose BKM	DRI CRI	DRI BKM
0.05	19.0840	66.6018	0.00649	0.01133
0.1	11.7051	35.6518	0.02225	0.03388
0.15	8.64838	24.2129	0.04768	0.06675
0.2	6.88614	18.0940	0.08465	0.11121
0.25	5.70480	14.2247	0.13599	0.16954
0.3	4.83992	11.5282	0.20576	0.24505
0.35	4.16856	9.52471	0.29972	0.34242
0.4	3.62499	7.96668	0.42616	0.46828
0.45	3.17046	6.71261	0.59722	0.63223
0.5	2.78040	5.67549	0.83128	0.84843
0.55	2.43834	4.79861	1.15708	1.13855
0.6	2.13260	4.04325	1.62154	1.53716
0.65	1.85451	3.38186	2.30555	2.10219
0.7	1.59727	2.79413	3.35840	2.93746

Fa	Dose CRI	Dose BKM	DRI CRI	DRI BKM
0.75	1.35511	2.26446	5.08148	4.24570
0.8	1.12264	1.78022	8.16348	6.47258
0.85	0.89388	1.33033	14.4926	10.7844
0.9	0.66045	0.90349	31.0622	21.2464
0.95	0.40509	0.48364	106.412	63.5234
0.97	0.28608	0.31004	255.553	138.474

DRI values calculated at experimental points

Fa	Dose CRI	Dose BKM	DRI CRI	DRI BKM
0.57	2.31222	4.48363	0.92489	0.89673
0.59	2.19129	4.18604	1.75303	1.67442
0.64	1.90826	3.50768	3.02899	2.78387
0.71	1.54780	2.68399	4.99291	4.32902
0.82	1.03108	1.59674	6.44422	4.98982

DRI Plot for Combo: CRIBKM (CRI+BKM [1:2])

Log(DRI) Plot for Combo: CRIBKM (CRI+BKM [1:2])

Isobologram for Combo: CRIBKM (CRI+BKM [1:2])

Polygonogram at Fa = 0.9

Summary Table

**Experiment Name:**	H2596 PLATE-1
**Date:**	10-28-13
**File Name:**	C:\Documents and Settings\idhanasingh\Desktop\Crizotinib BKM synergy\STEP-2 10-28-13\H2596 PLATE-1.cse
**Description**	SYNERGY STEP-2 BETWEEN CRI AND BKM

**Drug:**	CRIZOTINIB (CRI) [uM]
**Drug:**	BKM120 (BKM) [uM]
**Drug Combo:**	CRI-BKM120 (CRIBKM) (CRI+BKM [1:2])

Drug/Combo	Dm	m	r
CRI	2.78040	-1.5286	-0.9760
BKM	5.67549	-1.1957	-0.9387
CRIBKM	10.0342	-0.4344	-0.9447

CI values at:

Combo	ED50	ED75	ED90	ED95
CRIBKM	2.38161	0.43233	0.07926	0.02514

Data for Fa = 0.5

Drug/Combo	CI value	Dose CRI	Dose BKM
CRI		2.78040	
BKM			5.67549
CRIBKM	2.38161	3.34472	6.68944

Data for Fa = 0.75

Drug/Combo	CI value	Dose CRI	Dose BKM
CRI		1.35511	
BKM			2.26446
CRIBKM	0.43233	0.26668	0.53335

Data for Fa = 0.9

Drug/Combo	CI value	Dose CRI	Dose BKM
CRI		0.66045	
BKM			0.90349
CRIBKM	0.07926	0.02126	0.04252

Data for Fa = 0.95

Drug/Combo	CI value	Dose CRI	Dose BKM
CRI		0.40509	
BKM			0.48364
CRIBKM	0.02514	0.00381	0.00761

Data for Fa = 0.97

Drug/Combo	CI value	Dose CRI	Dose BKM
CRI		0.28608	
BKM			0.31004
CRIBKM	0.01113	0.00112	0.00224
