# Supplementary material for: PI3 Kinase Pathway and MET Inhibition is Efficacious in Malignant Pleural Mesothelioma
Source: Sci Rep. 2016 Sep 13;6:32992. doi: 10.1038/srep32992 (PMC5021085; doi:10.1038/srep32992)
Supplement: Supplementary Dataset 3 [file srep32992-s4.pdf]

CompuSyn Report

**Experiment Name:**	H2596 average between plate 1&2
**Date:**	9-2-13
**File Name:**	C:\Documents and Settings\idhanasingh\Desktop\CRIZOTINIB-GDC SYNERGY\9-3-13 STEP-2\H2596 AVERGE PLATE 1&2.cse
**Description**	synergy step-2 average of plates 1 and 2 H2596

Drug: CRIZOTINIB (CRI) [uM]
Drug: GDC0980 (GDC) [uM]
Drug Combo: CRI-GDC (CRIGDC) (CRI+GDC [1:1])

Data for Drug: CRI [uM]

Dose	Effect
3.0	0.09
1.5	0.19
0.75	0.28
0.38	0.49
0.19	0.99

5 data points entered.

X-int: -0.1287
Y-int: -0.2842 +/- 0.29244
m: -2.2094 +/- 0.66360
Dm: 0.74361
r: -0.8871

Data for Drug: GDC [uM]

Dose	Effect
3.0	0.15
1.5	0.17
0.75	0.22
0.38	0.34
0.19	0.53

5 data points entered.

X-int: -0.7858
Y-int: -0.5279 +/- 0.04880
m: -0.6717 +/- 0.11073

Dm: 0.16374
r: -0.9616

Data for Drug Combo: CRIGDC (CRI+GDC [1:1])

Dose A	Effect
3.0+	0.07
1.5+	0.1
0.75+	0.13
0.38+	0.2
0.19+	0.39

5 data points entered.

X-int: -0.8237
Y-int: -0.6082 +/- 0.04742
m: -0.7384 +/- 0.10324
Dm: 0.15008
r: -0.9719

Dose-Effect Curve

Median-Effect Plot

CI Data for Drug Combo: CRIGDC (CRI+GDC [1:1])

Fa	CI Value	Total Dose
0.05	1.74405	8.09397
0.1	1.07294	2.94220
0.15	0.84522	1.57242
0.2	0.73264	0.98108
0.25	0.66715	0.66451
0.3	0.62563	0.47281
0.35	0.59806	0.34708
0.4	0.57945	0.25990
0.45	0.56703	0.19695
0.5	0.55920	0.15008
0.55	0.55502	0.11437
0.6	0.55396	0.08667
0.65	0.55578	0.06490

Fa	CI Value	Total Dose
0.7	0.56055	0.04764
0.75	0.56865	0.03390
0.8	0.58102	0.02296
0.85	0.59967	0.01432
0.9	0.62957	0.00766
0.95	0.68776	0.00278
0.97	0.73545	0.00135

CI values for actual experimental points:

Total Dose	Fa	CI Value
6.0	0.07	1.64076
3.0	0.1	1.09402
1.5	0.13	0.69696
0.76	0.2	0.56754
0.38	0.39	0.80489

Combination Index Plot

Logarithmic Combination Index Plot

DRI Data for Drug Combo: CRIGDC (CRI+GDC [1:1])

Fa	Dose CRI	Dose GDC	DRI CRI	DRI GDC
0.05	2.81925	13.1156	0.69663	3.24084
0.1	2.01027	4.31224	1.36651	2.93130
0.15	1.63048	2.16575	2.07385	2.75466
0.2	1.39268	1.28950	2.83906	2.62873
0.25	1.22265	0.84029	3.67986	2.52907
0.3	1.09119	0.57803	4.61579	2.44511
0.35	0.98408	0.41151	5.67065	2.37127
0.4	0.89341	0.29943	6.87494	2.30419
0.45	0.81432	0.22075	8.26927	2.24166
0.5	0.74361	0.16374	9.90947	2.18204
0.55	0.67905	0.12146	11.8750	2.12399
0.6	0.61894	0.08954	14.2834	2.06635
0.65	0.56191	0.06515	17.3168	2.00791
0.7	0.50675	0.04638	21.2742	1.94727

Fa	Dose CRI	Dose GDC	DRI CRI	DRI GDC
0.75	0.45227	0.03191	26.6851	1.88262
0.8	0.39705	0.02079	34.5881	1.81124
0.85	0.33914	0.01238	47.3504	1.72844
0.9	0.27507	0.00622	71.8602	1.62429
0.95	0.19614	0.00204	140.961	1.46915
0.97	0.15419	9.26E-4	227.666	1.36787

DRI values calculated at experimental points

Fa	Dose CRI	Dose GDC	DRI CRI	DRI GDC
0.07	2.39779	7.70013	0.79926	2.56671
0.1	2.01027	4.31224	1.34018	2.87482
0.13	1.75799	2.77435	2.34399	3.69914
0.2	1.39268	1.28950	3.66494	3.39343
0.39	0.91049	0.31868	4.79205	1.67726

DRI Plot for Combo: CRIGDC (CRI+GDC [1:1])

Log(DRI) Plot for Combo: CRIGDC (CRI+GDC [1:1])

Isobologram for Combo: CRIGDC (CRI+GDC [1:1])

Polygonogram at Fa = 0.9

Summary Table

**Experiment Name:**	H2596 average between plate 1&2
**Date:**	9-2-13
**File Name:**	C:\Documents and Settings\idhanasingh\Desktop\CRIZOTINIB-GDC SYNERGY\9-3-13 STEP-2\H2596 AVERGE PLATE 1&2.cse
**Description**	synergy step-2 average of plates 1 and 2 H2596

**Drug:**	CRIZOTINIB (CRI) [uM]
**Drug:**	GDC0980 (GDC) [uM]
**Drug Combo:**	CRI-GDC (CRIGDC) (CRI+GDC [1:1])

Drug/Combo	Dm	m	r
CRI	0.74361	-2.2094	-0.8871
GDC	0.16374	-0.6717	-0.9616
CRIGDC	0.15008	-0.7384	-0.9719

CI values at:

Combo	ED50	ED75	ED90	ED95
CRIGDC	0.55920	0.56865	0.62957	0.68776

Data for Fa = 0.5

Drug/Combo	CI value	Dose CRI	Dose GDC
CRI		0.74361	
GDC			0.16374
CRIGDC	0.55920	0.07504	0.07504

Data for Fa = 0.75

Drug/Combo	CI value	Dose CRI	Dose GDC
CRI		0.45227	
GDC			0.03191
CRIGDC	0.56865	0.01695	0.01695

Data for Fa = 0.9

Drug/Combo	CI value	Dose CRI	Dose GDC
CRI		0.27507	
GDC			0.00622
CRIGDC	0.62957	0.00383	0.00383

Data for Fa = 0.95

Drug/Combo	CI value	Dose CRI	Dose GDC
CRI		0.19614	
GDC			0.00204
CRIGDC	0.68776	0.00139	0.00139

Data for Fa = 0.97

Drug/Combo	CI value	Dose CRI	Dose GDC
CRI		0.15419	
GDC			9.26E-4
CRIGDC	0.73545	6.77E-4	6.77E-4
