# Supplementary material for: PI3 Kinase Pathway and MET Inhibition is Efficacious in Malignant Pleural Mesothelioma
Source: Sci Rep. 2016 Sep 13;6:32992. doi: 10.1038/srep32992 (PMC5021085; doi:10.1038/srep32992)
Supplement: Supplementary Dataset 4 [file srep32992-s5.pdf]

# CompuSyn Report

| | |
|---|---|
| **Experiment Name:** | H513 PLATE-1 |
| **Date:** | 10-28-13 |
| **File Name:** | C:\Documents and Settings\idhanasingh\Desktop\Crizotinib BKM synergy\STEP-2 10-28-13\H513 PLATE-1.cse |
| **Description** | SYNERGY STEP-2 BETWEEN CRI AND BKM |

| | |
|---|---|
| **Drug:** | CRIZOTINIB (CRI) [uM] |
| **Drug:** | BKM120 (BKM) [uM] |
| **Drug Combo:** | CRIZO-BKM120 (CRIBKM) (CRI+BKM [4:1]) |

---

Data for Drug: CRI [uM]

| Dose | Effect |
|---|---|
| 5.0 | 0.72 |
| 2.5 | 0.73 |
| 1.25 | 0.76 |
| 0.625 | 0.91 |
| 0.313 | 0.97 |

5 data points entered.

| | |
|---|---|
| **X-int:** | 0.93461 |
| **Y-int:** | 0.86082 +/- 0.10273 |
| **m:** | -0.9211 +/- 0.23538 |
| **Dm:** | 8.60218 |
| **r:** | -0.9144 |

---

Data for Drug: BKM [uM]

| Dose | Effect |
|---|---|
| 1.25 | 0.78 |
| 0.625 | 0.88 |
| 0.313 | 0.88 |
| 0.156 | 0.9 |
| 0.078 | 0.9 |

5 data points entered.

| | |
|---|---|
| **X-int:** | 2.30520 |
| **Y-int:** | 0.68714 +/- 0.07055 |
| **m:** | -0.2981 +/- 0.10674 |

| | |
|---|---|
| **Dm:** | 201.929 |
| **r:** | -0.8498 |

Data for Drug Combo: CRIBKM (CRI+BKM [4:1])

| Dose A | Effect |
|---|---|
| 5.0+ | 0.68 |
| 2.5+ | 0.67 |
| 1.25+ | 0.68 |
| 0.625+ | 0.73 |
| 0.313+ | 0.74 |

5 data points entered.

| | |
|---|---|
| **X-int:** | 3.13542 |
| **Y-int:** | 0.39408 +/- 0.01833 |
| **m:** | -0.1257 +/- 0.03919 |
| **Dm:** | 1365.91 |
| **r:** | -0.8799 |

Dose-Effect Curve

Median-Effect Plot

CI Data for Drug Combo: CRIBKM (CRI+BKM [4:1])

| Fa | CI Value | Total Dose |
|---|---|---|
| 0.05 | 7.76E10 | 2.04E13 |
| 0.1 | 4.573E8 | 5.34E10 |
| 0.15 | 1.905E7 | 1.346E9 |
| 0.2 | 1740401 | 8.426E7 |
| 0.25 | 241225. | 8542307 |
| 0.3 | 42936.0 | 1156596 |
| 0.35 | 8957.38 | 188129. |
| 0.4 | 2068.26 | 34393.0 |
| 0.45 | 507.699 | 6742.51 |
| 0.5 | 128.382 | 1365.91 |
| 0.55 | 32.5355 | 276.709 |
| 0.6 | 8.04443 | 54.2470 |
| 0.65 | 1.88455 | 9.91722 |

| Fa | CI Value | Total Dose |
|---|---|---|
| 0.7 | 0.40383 | 1.61311 |
| 0.75 | 0.07558 | 0.21841 |
| 0.8 | 0.01157 | 0.02214 |
| 0.85 | 0.00131 | 0.00139 |
| 0.9 | 9.03E-5 | 3.49E-5 |
| 0.95 | 1.97E-6 | 9.15E-8 |
| 0.97 | 1.58E-7 | 1.33E-9 |

CI values for actual experimental points:

| Total Dose | Fa | CI Value |
|---|---|---|
| 6.25 | 0.68 | 1.39520 |
| 3.125 | 0.67 | 0.66028 |
| 1.5625 | 0.68 | 0.34880 |
| 0.78125 | 0.73 | 0.23569 |
| 0.39125 | 0.74 | 0.12622 |

---

Combination Index Plot

---

Logarithmic Combination Index Plot

---

DRI Data for Drug Combo: CRIBKM (CRI+BKM [4:1])

| Fa | Dose CRI | Dose BKM | DRI CRI | DRI BKM |
|---|---|---|---|---|
| 0.05 | 210.363 | 3936945 | 1.3E-11 | 9.65E-7 |
| 0.1 | 93.4637 | 320989. | 2.19E-9 | 3.00E-5 |
| 0.15 | 56.5597 | 67992.6 | 5.25E-8 | 2.52E-4 |
| 0.2 | 38.7502 | 21134.4 | 5.75E-7 | 0.00125 |
| 0.25 | 28.3548 | 8050.91 | 4.15E-6 | 0.00471 |
| 0.3 | 21.5837 | 3464.87 | 2.33E-5 | 0.01498 |
| 0.35 | 16.8460 | 1611.09 | 1.12E-4 | 0.04282 |
| 0.4 | 13.3596 | 786.955 | 4.86E-4 | 0.11441 |
| 0.45 | 10.6962 | 395.886 | 0.00198 | 0.29358 |
| 0.5 | 8.60218 | 201.929 | 0.00787 | 0.73917 |
| 0.55 | 6.91813 | 102.998 | 0.03125 | 1.86112 |
| 0.6 | 5.53891 | 51.8142 | 0.12763 | 4.77577 |
| 0.65 | 4.39258 | 25.3092 | 0.55366 | 12.7602 |
| 0.7 | 3.42840 | 11.7682 | 2.65667 | 36.4769 |

| Fa | Dose CRI | Dose BKM | DRI CRI | DRI BKM |
|---|---|---|---|---|
| 0.75 | 2.60971 | 5.06470 | 14.9359 | 115.946 |
| 0.8 | 1.90961 | 1.92934 | 107.806 | 435.681 |
| 0.85 | 1.30831 | 0.59970 | 1180.19 | 2163.90 |
| 0.9 | 0.79173 | 0.12703 | 28337.9 | 18187.0 |
| 0.95 | 0.35176 | 0.01036 | 4808076 | 566268. |
| 0.97 | 0.19750 | 0.00174 | 1.855E8 | 6538990 |

DRI values calculated at experimental points

| Fa | Dose CRI | Dose BKM | DRI CRI | DRI BKM |
|---|---|---|---|---|
| 0.68 | 3.79482 | 16.1056 | 0.75896 | 12.8845 |
| 0.67 | 3.98736 | 18.7670 | 1.59494 | 30.0272 |
| 0.68 | 3.79482 | 16.1056 | 3.03585 | 51.5378 |
| 0.73 | 2.92163 | 7.17900 | 4.67460 | 45.9456 |
| 0.74 | 2.76321 | 6.04302 | 8.82815 | 77.2271 |

---

DRI Plot for Combo: CRIBKM (CRI+BKM [4:1])

---

Log(DRI) Plot for Combo: CRIBKM (CRI+BKM [4:1])

---

Isobologram for Combo: CRIBKM (CRI+BKM [4:1])

---

Polygonogram at Fa = 0.9

---

# Summary Table

| | |
|---|---|
| **Experiment Name:** | H513 PLATE-1 |
| **Date:** | 10-28-13 |
| **File Name:** | C:\Documents and Settings\idhanasingh\Desktop\Crizotinib BKM synergy\STEP-2 10-28-13\H513 PLATE-1.cse |
| **Description** | SYNERGY STEP-2 BETWEEN CRI AND BKM |

| | |
|---|---|
| **Drug:** | CRIZOTINIB (CRI) [uM] |
| **Drug:** | BKM120 (BKM) [uM] |
| **Drug Combo:** | CRIZO-BKM120 (CRIBKM) (CRI+BKM [4:1]) |

| Drug/Combo | Dm | m | r |
|---|---|---|---|
| CRI | 8.60218 | -0.9211 | -0.9144 |
| BKM | 201.929 | -0.2981 | -0.8498 |
| CRIBKM | 1365.91 | -0.1257 | -0.8799 |

CI values at:

| Combo | ED50 | ED75 | ED90 | ED95 |
|---|---|---|---|---|
| CRIBKM | 128.382 | 0.07558 | 9.03E-5 | 1.97E-6 |

Data for Fa = 0.5

| Drug/Combo | CI value | Dose CRI | Dose BKM |
|---|---|---|---|
| CRI | | 8.60218 | |
| BKM | | | 201.929 |
| CRIBKM | 128.382 | 1092.73 | 273.182 |

Data for Fa = 0.75

| Drug/Combo | CI value | Dose CRI | Dose BKM |
|---|---|---|---|
| CRI | | 2.60971 | |
| BKM | | | 5.06470 |
| CRIBKM | 0.07558 | 0.17473 | 0.04368 |

Data for Fa = 0.9

| Drug/Combo | CI value | Dose CRI | Dose BKM |
|---|---|---|---|
| CRI | | 0.79173 | |
| BKM | | | 0.12703 |
| CRIBKM | 9.03E-5 | 2.79E-5 | 6.98E-6 |

Data for Fa = 0.95

| Drug/Combo | CI value | Dose CRI | Dose BKM |
|---|---|---|---|
| CRI | | 0.35176 | |
| BKM | | | 0.01036 |
| CRIBKM | 1.97E-6 | 7.32E-8 | 1.83E-8 |

Data for Fa = 0.97

| Drug/Combo | CI value | Dose CRI | Dose BKM |
|---|---|---|---|
| CRI | | 0.19750 | |
| BKM | | | 0.00174 |
| CRIBKM | 1.58E-7 | 1.06E-9 | 2.7E-10 |
